# Publisher Correction: Sudden quench of harmonically trapped mass-imbalanced fermions

**DOI:** 10.1038/s41598-023-29085-y

**Published:** 2023-02-07

**Authors:** Dillip K. Nandy, Tomasz Sowiński

**Affiliations:** 1 Center for Theoretical Physics of Complex Systems, Institute for Basic Science (IBS), Daejeon, 34126 Korea; 2 Institute of Physics, Polish Academy of Sciences, Aleja Lotników 32/46, 02668 Warsaw, Poland

Correction to: *Scientific Reports* https://doi.org/10.1038/s41598-022-24228-z, published online 16 November 2022

The original version of this Article contained an error in Reference 10, which was incorrectly given as:

Das, S. Particle scattering by harmonically trapped quantum gases in an artificial magnetic field. Phys. B Condens. Matter **635**, 413833. (2022).

The correct reference is listed below:

Das, S. Particle scattering by harmonically trapped quantum gases in an artificial magnetic field. *Phys. B Condens. Matter* **635**, 413833. https://doi.org/10.1016/j.physb.2022.413833 (2022).

The original Article has been corrected.

